# Progerin mRNA Is Associated with Smoking and Signs of Increased Microvascular Damage in Patients with Diabetic Macular Edema

**DOI:** 10.3390/ijms26052099

**Published:** 2025-02-27

**Authors:** Marc-Michael Zaruba, Reinhard Angermann, Simon Staggl, Vivek Jeyakumar, Sofia Mair, Victoria Stöckl, Julia Neyer, Thomas Maurer, Maria Ungericht, Jasmina Gavranovic-Novakovic, Axel Bauer, Claus Zehetner, Moritz Messner

**Affiliations:** 1 Department of Internal Medicine III, Cardiology and Angiology, Medical University Innsbruck, 6020 Innsbruck, Austriavivek.jeyakumar@i-med.ac.at (V.J.); jasmina.gavranovic-novakovic@i-med.ac.at (J.G.-N.);; 2 Department of Ophthalmology, Medical University Innsbruck, 6020 Innsbruck, Austria; 3 Department of Ophthalmology, LK Mistelbach/Gänserndorf, 2130 Mistelbach, Austria

**Keywords:** Hutchinson–Gilford Syndrome, diabetic retinopathy, progerin, lamin A, alternative splicing

## Abstract

The premature aging disease Hutchinson–Gilford Syndrome (HGPS) is caused by defined mutations in the LMNA gene, resulting in the activation of a cryptic splice donor site, which leads to a defective truncated prelamin A protein called progerin. Notably, progerin expression has also been detected in non-mutated healthy individuals, and therefore, its involvement in the physiological aging process has been widely discussed. Since diabetes mellitus is associated with premature aging and increased cardiovascular mortality, we aimed to investigate the role of progerin expression in patients with diabetic retinopathy (DR). mRNA expression of progerin was analyzed in blood samples from 140 patients with DR who received anti-vascular endothelial growth factor (VEGF) therapy. Progerin mRNA levels were significantly lower in female compared to male patients (*n* = 42 vs. *n* = 98; 0.67 ± 0.19 vs. 0.89 ± 0.51, *p* = 0.006) and higher in patients with non-proliferative (NP)DR (*n* = 87 vs. *n* = 53; 0.9 ± 0.51 vs. 0.71 ± 0.29, *p* = 0.013) compared to those with proliferative (P)DR. Additionally, a positive correlation was found between progerin mRNA expression and the number of intravitreal anti-VEGF applications (*n* = 139, r = 0.21, *p* = 0.015), central macula thickness (CMT), (*n* = 137, r = 0.18, *p* = 0.036) and nicotine consumption (*n* = 105, r = 0.235, *p* = 0.002). The nuclear localization and significant upregulation of progerin mRNA and protein levels in dermal fibroblasts from HGPS donors emphasize its role in cellular aging mechanisms. Progerin mRNA levels were higher in patients with NPDR. CMT, number of intravitreal anti-VEGF therapy treatments, and cigarette consumption were positively related to progerin mRNA, suggesting an association with disease progression and premature aging.

## 1. Introduction

Hutchinson–Gilford Progeria Syndrome (HGPS) is a laminopathy characterized by premature aging. This includes the early onset of hair loss, joint abnormalities, ocular diseases, high blood pressure, and atherosclerosis, causing myocardial infarction and stroke, with fatal consequences until the age of 25 [1].

**Figure 1:**
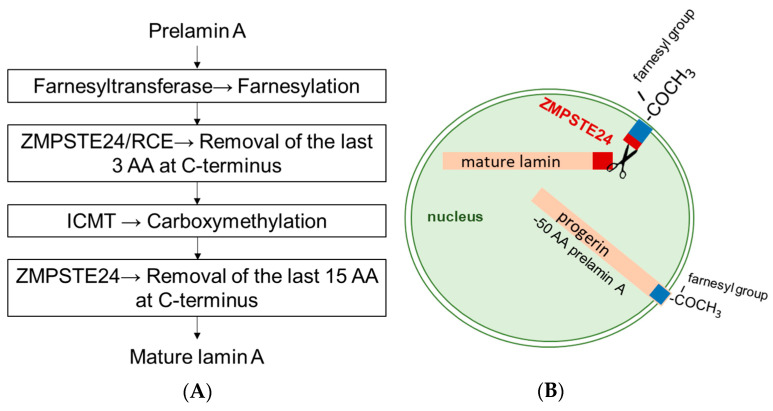
The first three steps of post-transcriptional modification of prelamin A to mature lamin (**A**) include farnesylation of the cysteine in the C-terminal CaaX motif (C, cysteine; a, aliphatic; and X, any amino acid), proteolytic cleavage of the aaX-terminal tripeptide, and methylation of the farnesylated cysteine. In the fourth and final step of post-transcriptional modification, the protein needs to lose its farnesyl modification through the proteolytic cleavages by the zinc metalloproteinase ZMSPTE24 to yield mature lamin A. Because the cleavage site for ZMPSTE24 is lost (**B**) in the mutant protein, progerin remains permanently farnesylated, causing a tight association with the nuclear envelope and leading to numerous nuclear envelope abnormalities.

HGPS is caused by mutations affecting the encoding and processing of lamin A (LMNA). Lamins are type V intermediate filaments that are important for the nuclear structure and transcriptional regulation [2]. In about 90% of the cases, a point mutation (LMNA 1824 C>T, G608G) causes a frameshift that leads to alternative splicing, ultimately producing a truncated form of prelamin A [3]. This form of prelamin A lacks the cleavage site for a zinc metalloprotease (ZMPSTE24). The lipid (farnesyl) anchor on prelamin A that fixes the protein to the inner nuclear membrane is normally cleaved off by ZMPSTE24 as the last step of its post-translational modification. Failure to cleave the farnesyl residue leads to an accumulation of prelamin A in the inner nuclear membrane (Figure 1) and disruption of the mechanical stability of the cell. This leads to a higher rate of cell death and accelerated aging.

In addition to other manifestations, children with HGPS are prone to age-related ophthalmic diseases such as senile ectropion, madarosis, corneal dryness, dry eye syndrome, and nocturnal lagophthalmos with consecutive exposure keratopathy. Moreover, there are reports describing an association between HGPS and retinal arteriolar narrowing, tortuosity, and retinal angiosclerosis. Pathological changes of retinal vessels are common issues seen in degenerative macular diseases, such as diabetic retinopathy (DR) [4].

Several groups have reported that progerin is not only expressed in cells of individuals with a specific genetic mutation but also occurs in otherwise healthy individuals through alternative splicing. This prompted a comprehensive debate about the potential role of alternatively spliced progerin in the physiological process of aging [5,6]. For example, Clintock et al. postulated that small amounts of progerin mRNA are produced in skin cells by alternative splicing, resulting in a gradual accumulation of progerin protein over time. They concluded that the quantity of accumulated progerin would be suitable as a biomarker for cellular aging in human skin [7]. Other studies indicate that smoking exacerbates microvascular damage in patients with diabetes, increasing the risk of developing DME. Chronic smoking impairs retinal blood flow (RBF) and elevates vascular resistance, which in turn leads to further microvascular complications, particularly in individuals with type 2 diabetes [8]. Additionally, smoking has been linked to the progression of DR, with studies indicating that smokers face a significantly higher risk of proliferative diabetic retinopathy (PDR) [9].

DR is a condition in which diabetes-related changes in the vascular system can be directly visualized. It is a vision-threatening disease associated with increased inflammatory markers, leading to vascular leakage caused by retinal microangiopathy. In HGPS patients, the vascular system is similarly affected by accelerated aging, resulting in death from heart attacks or strokes at a young age. Oxidative stress can cause a loss of pericytes, impairing blood vessel function and the blood–retina barrier, like in diabetic macular edema (DME). Hyperglycemia activates the polyol pathway and turns glucose into sorbitol by aldose reductase under the consumption of NADPH, thus increasing oxidative stress. Recent research suggests that using aldose reductase inhibitors may help reduce these harmful effects, which could be a promising direction for future treatments [10].

We investigated DR because understanding the molecular mechanisms underlying this condition can provide crucial insights into the effects of premature aging, potentially leading to novel therapeutic approaches. Specifically, we aim to explore the association between progerin mRNA levels and various clinical parameters in a cohort of patients with DR to determine whether there is a link between accelerated aging mechanisms and the severity of DR. The fibroblast experiments were conducted to investigate the cellular localization and expression levels of progerin, providing a mechanistic link between its role in nuclear architecture disruption and premature aging processes.

In this study, we investigated the association between progerin mRNA levels and clinical parameters in patients with DR. In particular, we examined whether progerin mRNA expression correlates with the severity of DR (non-proliferative [NP]DR versus proliferative [P]DR), central macula thickness (CMT), the frequency of intravitreal anti-vascular endothelial growth factor (VEGF) therapy, and smoking status.

## 2. Results

**Figure 2:**
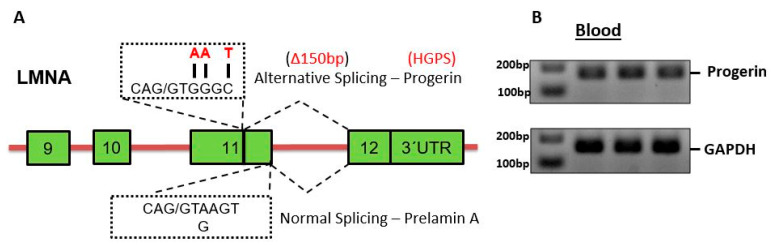
(**A**) Schematic illustration showing the canonical donor splice sites at the terminus of exon 11, which is necessary for the normal splicing of LMNA, and the cryptic splice site, along with three prevalent mutations within exon 11, which result in the aberrant splicing event, producing progerin mRNA. (**B**) RT-PCR performed on three human blood samples, employing primers spanning exon 11 to exon 12 to concurrently detect progerin mRNA. GAPDH mRNA was used as an internal control.

As illustrated in Figure 2A, we designed specific primers for the detection of the 150 bp long progerin splice product. Utilizing these primers, we were able to clearly detect progerin splice variants in samples drawn from patients with DR.

**Figure 3:**
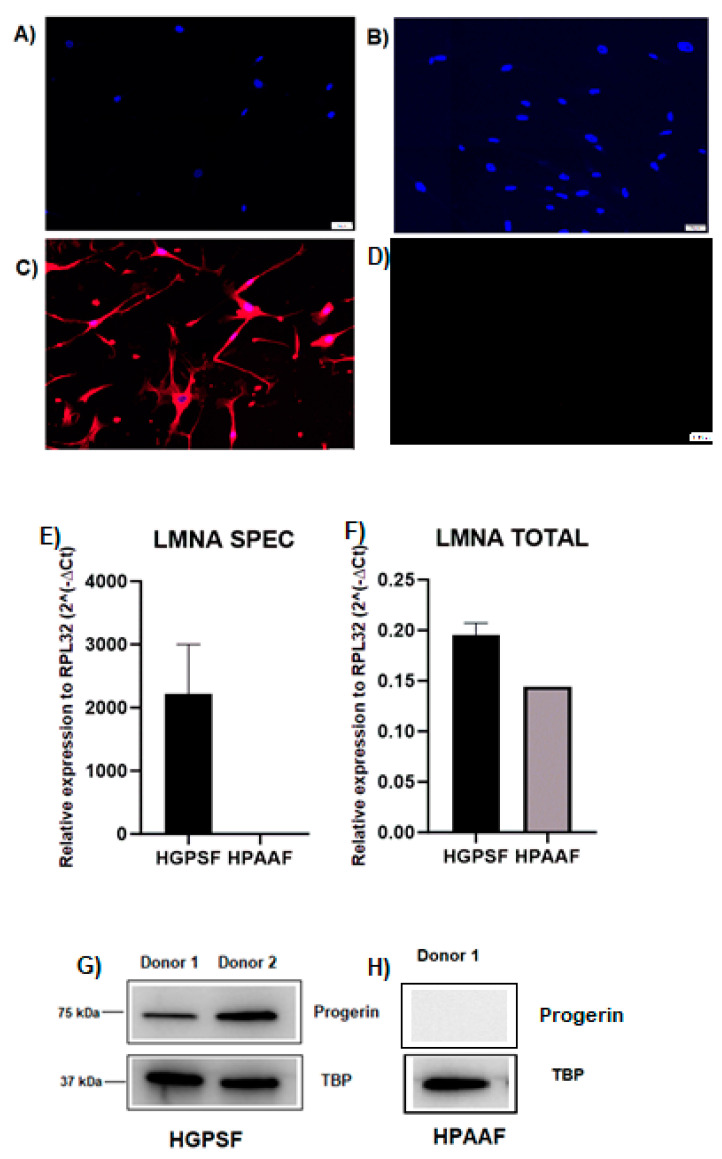
Progerin expression in dermal fibroblasts from HGPS donors. (**A**,**B**) DAPI staining illustrating nuclear morphology in dermal fibroblasts from HGPS patients (**A**) and control human pulmonary artery adventitial fibroblasts (HPAAF) (**B**). (**C**,**D**) Progerin immunostaining (red fluorescence) showing strong nuclear envelope localization in HGPS fibroblasts (**C**), with no detectable signal observed in HPAAF controls, scale bar represents 100 µm (**D**). (**E**,**F**) RT-qPCR analysis of progerin mRNA expression in HGPS fibroblasts compared to HPAAF controls. (**E**) LMNA SPEC mRNA expression, normalized to RPL32, is significantly upregulated in HGPS fibroblasts. (**F**) LMNA TOTAL mRNA expression, also normalized to RPL32, shows increased levels in HGPS fibroblasts. Data are presented as mean ± SD. (**G**,**H**) Western blot analysis of progerin protein levels. (**G**) Robust progerin expression is detected in fibroblasts from two HGPS donors, with TBP as a loading control. (**H**) Progerin is absent in healthy control fibroblasts (HPAAF), confirming specificity.

The expression of progerin in dermal fibroblasts from HGPS donors was compared to that of healthy controls (HPAAF) (Figure 3). Immunostaining (Figure 3A–C) highlights strong nuclear localization of progerin in HGPS fibroblasts, with red fluorescence indicating progerin accumulation, predominantly at the nuclear envelope. In contrast, no detectable progerin signal was observed in healthy controls. RT-qPCR analysis (Figure 3D,E) demonstrates a significant upregulation of progerin mRNA levels in HGPS fibroblasts, both for LMNA SPEC and LMNA TOTAL, normalized to the RPL32 housekeeping gene. Western blot analysis (Figure 3F,G) further confirms these findings, showing high levels of progerin protein in HGPS fibroblasts and its absence in HPAAF controls, with TBP serving as an internal loading control. These data provide compelling evidence of progerin’s upregulation at both the mRNA and protein levels, reinforcing its role in nuclear disruption and cellular aging in HGPS fibroblasts.

Of the total 140 patients consecutively included in this study, the mean (SD) age was 66 (12) years, and the median (IQR) was 67 (59–75). Forty-two patients (30%) were female, and twenty-eight (20%) patients suffered from type I diabetes. Seventy patients (50%) were pseudophakic in both eyes and eighty-two (59%) patients were pseudophakic in at least one eye. The majority of patients (*n* = 112, 80%) required insulin treatment for diabetes. Forty-five patients stated that they were current smokers, and the entire cohort had a median (IQR) of 10 (0–38) pack-years. Patients had been receiving treatment for DR at the Ophthalmological Department for a median (IQR) of 3.6 (1.3–6.9) years and received a median of 7 (2–24) anti-VEGF injections. Further baseline characteristics are shown in Table 1. Fifty-three patients (38%) showed signs of PDR. Patients with NPDR were significantly older [mean 69 ± 10, median 71 (62–77) vs. mean 61 ± 12, median 62 (54–72) years; *p* < 0.001] and received significantly more anti-VEGF injections (10, 4–34 vs. 5, 2–11; *p* = 0.035) compared to patients with PDR. The demographic and clinical differences between patients with NPDR and PDR are presented in detail in Table 2.

**Figure 4:**
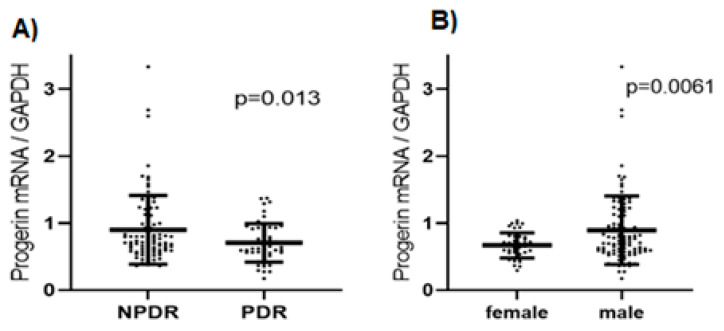
(**A**) Scatterplot diagram showing all individual data points; lines show mean values ± SD of progerin mRNA related to the reference gene GAPDH between the NPDR (*n* = 87) and PDR (*n* = 53) (*p* = 0.013) groups. (**B**) Scatterplot diagram showing all individual data points; lines show mean values ± SD of progerin mRNA in female (*n* = 42) and male patients (*n* = 98) (*p* = 0.006 between groups).

As shown in Figure 4, progerin mRNA levels were significantly upregulated in blood samples of individuals with NPDR (*n* = 87; 0.9 ± 0.51 vs. 0.71 ± 0.29, *p* = 0.013) compared to the PDR group (*n* = 53) (Figure 4A). Furthermore, we observed significantly lower progerin levels (Figure 4B) in female patients (*n* = 42) than in male patients (*n* = 98; 0.67 ± 0.19 vs. 0.89 ± 0.51, *p* = 0.006).

We further performed linear regression analyses of the total population based on Pearson’s correlation to calculate whether progerin mRNA expression correlates to the number of intravitreal operative injection of medication (IVOM) anti-VEGF applications (*n* = 139), macula thickness (*n* = 137), and consumption of cigarettes (*n* = 105).

**Figure 5:**
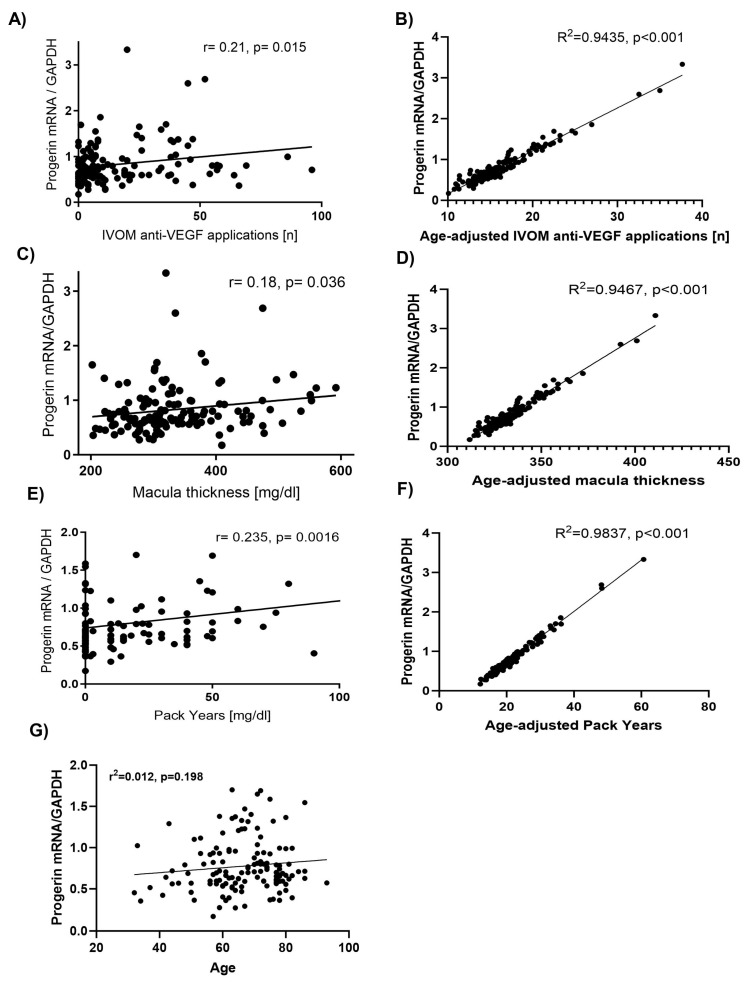
Scatter plots depicting the relationship between progerin mRNA/GAPDH expression and various clinical parameters, with and without age adjustment. (**A**) Intravitreal anti-VEGF injections (IVOM) as a function of progerin mRNA/GAPDH expression. A weak positive correlation was observed (r = 0.21, *p* = 0.015). (**B**) Age-adjusted relationship between IVOM anti-VEGF injections and progerin mRNA/GAPDH expression. The association strengthened substantially after age adjustment (R^2^ = 0.9435, *p* < 0.001), highlighting age as a significant confounding factor. (**C**) Macula thickness (µm) as a function of progerin mRNA/GAPDH expression, showing a weak positive correlation (r = 0.18, *p* = 0.036). (**D**) Age-adjusted relationship between macula thickness and progerin mRNA/GAPDH expression. The association became much stronger after age adjustment (R^2^ = 0.9467, *p* < 0.001), further emphasizing the confounding effect of age. (**E**) Correlation between pack-years of smoking and progerin mRNA/GAPDH expression, revealing a weak positive correlation (r = 0.235, *p* = 0.0016). (**F**) Age-adjusted relationship between pack-years of smoking and progerin mRNA/GAPDH expression. The correlation became very strong after adjusting for age (R^2^ = 0.9837, *p* < 0.001), again demonstrating the influence of age as a confounder. (**G**) Progerin mRNA/GAPDH expression as a function of age. A weak non-significant association was observed (R^2^ = 0.012, *p* = 0.198), indicating that progerin mRNA expression is not strongly correlated with chronological age alone.

We observed a positive correlation between progerin mRNA levels in patients’ blood and the number of IVOM anti-VEGF applications (r = 0.21, *p* = 0.015) (Figure 5A). After adjusting for age, the correlation remained significant (r = 0.94, *p* < 0.001) (Figure 5B). Similarly, CMT (r = 0.18, *p* = 0.036) (Figure 5C) and nicotine consumption (r = 0.235, *p* = 0.002) (Figure 5E) showed positive correlations with progerin mRNA levels, which remained significant after adjusting for age (CMT: r = 0.94, *p* < 0.001; nicotine consumption: r = 0.98, *p* < 0.001) (Figure 5D,F). These findings suggest that the relationship between progerin mRNA levels and these clinical parameters is not solely driven by age.

## 3. Discussion

Low levels of the lamin A splice variant progerin, associated with premature aging in HGPS, are found in normal vascular cells during aging, suggesting a role in physiological vascular aging. Progerin has been shown to cause a loss of endothelial integrity and is likely to be a determinant of cellular senescence and dysfunction [11]. The presence of progerin in retinal vascular cells can aggravate the vascular pathology observed in diabetic retinopathy [12]. To deepen our understanding of the role of progerin in premature vascular pathology, we analyzed the expression of progerin in a patient cohort with DM and DR.

DME and PDR are common complications associated with DM, contributing to severe visual impairment, one of the most feared complications from a patient’s perspective [13].

In previously published work, we found a positive correlation of progerin mRNA with markers of systemic inflammation [14]. In the state of chronic hyperglycemia in poorly controlled diabetes, metabolic intracellular pathways are induced, leading to reactive oxygen species, increased expression of inflammatory markers, and the initiation of an inflammatory cascade [15]. The increased oxidative stress consecutively leads to a loss of pericytes, endothelial cells, and loss of integrity of proteins of the tight junctions, resulting in endothelial dysfunction and a state known as degenerative microangiopathy [16]. Similarly, in HGPS vascular aging, severe atherosclerosis and obstruction occur independently of classical risk factors such as hypertension, smoking, and hypercholesterolemia. Therefore, HGPS may provide an opportunity to discover new factors contributing to the vascular diseases of aging.

Interestingly, we found a significant correlation between progerin mRNA levels and heavy smoking in our study cohort. Smoking is known to trigger endothelial dysfunction and generalized vascular inflammation [17]. The findings from dermal fibroblasts further support the role of progerin in premature aging and vascular damage. Elevated levels of progerin mRNA and protein in HGPS fibroblasts align with our observations in diabetic retinopathy patients, suggesting that comparable molecular mechanisms could be driving this process. The strong nuclear localization of progerin in fibroblasts highlights its disruptive effect on the nuclear architecture, which has been previously associated with cellular dysfunction and aging [18,19]. These in vitro findings support the idea that progerin could be a key mediator of vascular complications in diabetes, particularly in individuals with heavy smoking habits or other risk factors for accelerated aging. The consistent upregulation of progerin across different cell types and tissues suggests its broader relevance as a marker of cellular senescence, further linking premature aging mechanisms observed in HGPS to microvascular damage seen in diabetic retinopathy. Smoking, diabetes mellitus (DM), and HGPS are accompanied by accelerated vascular aging and enhanced atherosclerosis. Olive et al. showed that progerin expression contributes to vascular pathology in HGPS, leading to endothelial dysfunction and atherosclerosis. This aligns with our observation of increased progerin mRNA levels being associated with microvascular damage in DR patients [20].

The relationship between smoking and the development of DME remains a topic of ongoing debate. While smoking is widely recognized as a risk factor for numerous vascular diseases, its role in DME appears to be less clear. Thomson et al. recently suggested that smoking may, counterintuitively, reduce the risk of DME in patients with diabetes [21]. In contrast, Kramer et al. found that smoking is associated with an increased risk of DME, specifically in patients with type 1 diabetes mellitus [22]. Additionally, Al Saad et al. investigated the impact of smoking on anti-vascular endothelial growth factor (anti-VEGF) therapy outcomes, further adding to the complexity of this discussion [23].

Our findings do not support a direct correlation between smoking and central macular thickness (CMT) in our cohort, where only 20% of patients had type 1 diabetes. In the overall study population, no significant association between smoking and CMT was observed (OD r = −0.037, *p* = 0.722; OS r = 0.143, *p* = 0.161). Given the conflicting evidence, further studies with larger sample sizes are necessary to clarify whether smoking acts as a protective or detrimental factor in DME development.

Sex-related differences in progerin expression may be influenced by hormonal factors, particularly estrogen. Estrogen can influence the expression and activity of splicing factors, including SR proteins. Elhasnaoui et al. have shown that estrogen receptor α (ERα) signaling can regulate alternative splicing, even in the absence of ligands [24]. The depletion of ERα has been associated with changes in the expression of numerous RNA-binding proteins, including splicing factors involved in translation, ribonucleoprotein complex assembly, and 3′-end processing. This suggests that ERα plays a role in maintaining cellular homeostasis by regulating gene expression at the alternative splicing level [25]. Despite the lack of a statistically significant linear relationship between chronological age and progerin mRNA across the cohort, aligning with the complex and heterogeneous nature of aging, subgroup analysis unveiled a trend towards elevated progerin mRNA in older groups. This divergence emphasizes the greater relevance of biological age, potentially reflecting cumulative cellular stress, metabolic shifts, and inflammatory processes. The significant intragroup variability observed warrants focused investigation to determine the biological factors driving these age-related differences. Future studies should prioritize an in-depth analysis of these subgroups to define these underlying mechanisms.

Inflammation, in turn, is thought to play a decisive role in the development of the latter. Inflammatory cells produce a variety of mediators in response to smoking. Numerous studies have investigated the association between smoking and increased levels of inflammatory markers and cytokines, such as tumor necrosis factor alpha (TNF-α), interleukin (IL)-1, IL-6, and IL-8, as well as decreased levels of anti-inflammatory cytokines, such as IL-10 [26]. Systemic inflammation, in turn, was positively correlated with progerin mRNA levels in a previous study and plays a central role in the pathogenesis of degenerative microangiopathy and the development of DME [27]. It is indeed conceivable that smoking and, consequently, activated inflammatory processes lead to an increased cell turnover, thereby increasing the general expression of lamin A/C and its alternative splicing product progerin. To our knowledge, our data show, for the first time, an association between progerin mRNA in patients with DM and severe nicotine abuse, which may lead to vascular aging and consequently limit their life expectancy. Elevated CRP levels, indicative of systemic inflammation, were associated with increased progerin expression, reinforcing the link between vascular inflammation and DME progression. Further investigation is needed to determine whether targeting this pathway could have clinical benefits in the context of DME.

In DR, the cumulative loss of cell arrangement causes a hyperpermeability of the retinal vessels and thus increased vascular leakage into the extracellular space. This extravasation causes DME, which can occur at any stage of DR [28]. McClintock et al. found that progerin levels are elevated in individuals with diabetes, correlating with increased oxidative stress and inflammation. This supports our finding of higher progerin mRNA levels in patients with non-proliferative diabetic retinopathy (NPDR) and suggests a possible link between diabetes-related vascular complications and progerin expression [29].

The rising incidence of DM and patients with DR has become a major public health concern, representing the most common reason for visual loss among people aged under 60 years in industrialized countries [30]. Another notable finding of this study is the correlation between an increased expression of progerin mRNA and the number of anti-VEGF injections the patients received as part of the therapy for DME prior to blood sample collection. This suggests that progerin relates to disease progression, requiring anti-VEGF therapy. Additionally, we observed a correlation with CMT, which may be affected by the intensity of vascular leakage. Intriguingly, in our cohort, the expression of progerin mRNA was significantly higher in patients with NPDR compared to patients with PDR, which might be explained by the significantly increased age in this population, supporting our assumption that progerin is a marker for aging. However, the presence of DME does not only depend on current models trying to stage DR. In a study with 5 years of follow-up, Mosfhegi et al. described that patients suffering from NPDR were much more likely to develop DME than patients suffering from PDR [28].

Pathways of cellular senescence are activated in endothelial cells in the diabetic retina. Pharmacological elimination of senescent cells in a diabetic mouse model reduced retinal vascular leakage and preserved function. A phase 1 trial of UBX1325 (foselutoclax), a BCL-xL inhibitor, in advanced DME patients showed it was safe and well-tolerated [29].

Taken together, the findings of increased progerin mRNA in patients with NPDR, higher CMT, and a higher number of anti-VEGF injections indicate an association between the level of microvascular damage and disease progression/severity with the systemic expression of progerin mRNA. A study by Avery et al. highlights the role of anti-VEGF therapy in managing DME and its impact on vascular permeability and integrity. Our observation that higher progerin levels correlate with the number of intravitreal anti-VEGF applications suggests a relationship between VEGF therapy, vascular damage, and progerin expression [30].

In general, the question of commonalities between HGPS and natural aging remains. Moreover, the reason for the existence of such a cryptic splicing site still appears to be unclear. Olive M et al. found concordance between many aspects of cardiovascular pathology in both HGPS and geriatric patients. HGPS generates a more prominent adventitial fibrosis than typical cardiovascular diseases. Vascular progerin generation in young non-HGPS individuals, which significantly increases throughout life, strongly suggests that progerin has a role in cardiovascular aging of the general population [20].

While this study included patients with varied backgrounds, including those using insulin therapy, we did not perform a detailed subgroup analysis to specifically evaluate the impact of insulin usage on progerin mRNA levels. Future research should consider investigating these effects to better understand the potential influences of diabetes management therapies on progerin expression. This study did not include treatment-naïve control groups, which represents a limitation. Anti-VEGF therapy could potentially modify molecular events in retinal vessels, including progerin expression. Future studies should include treatment-naïve patients to better assess the baseline expression of progerin mRNA in diabetic retinopathy and its progression.

Aging-related tissue functional decline is linked to the onset of metabolic diseases. The LMNA gene exhibits dual roles in energy metabolism and lifespan regulation. Toledo et al. demonstrated that exclusive expression of lamin C (LMNA LCS/LCS mice) confers protection against glucose intolerance by inducing a β-cell–adaptive transcriptional response under metabolic stress [31]. This positions LMNA gene processing as a potential therapeutic target for diabetes management. Other animal models provide crucial insights into the mechanisms underlying diabetic retinopathy and DME. The streptozotocin (STZ)-induced diabetic mouse model has been widely used to study hyperglycemia-driven vascular dysfunction, while the Ins2Akita mouse model mimics genetic diabetes-associated retinal changes [32]. Oxygen-induced retinopathy (OIR) models have also provided valuable data on retinal neovascularization [33]. Future investigations should clarify whether strategies targeting LMNA splicing influence systemic diseases such as diabetes broadly or if a direct causal relationship leading to DME exists.

Owing to its nature as a cohort study, our trial lacks a control group that would allow for the unequivocal comparison of progerin levels. While our findings link smoking and progerin, the underlying mechanisms remain unclear. Future investigations should assess expression levels and post-translational modifications such as phosphorylation, methylation, and subcellular localization of key splicing factors known to regulate LMNA exon 11 inclusion/exclusion in cells exposed to cigarette smoke extract (CSE) or specific smoking components like nicotine.

## 4. Materials and Methods

### 4.1. Subjects

For this cross-sectional analysis, we consecutively recruited 140 patients receiving therapy for DR between December 2020 and March 2021 at the Ophthalmology Department of the Medical University of Innsbruck, Austria.

Patients diagnosed with clinically significant DME who were indicated for and treated with intravitreal anti-VEGF therapy were consecutively recruited for blood sample collection as part of a biobank initiative for degenerative macular diseases. Clinically significant DME was defined as retinal thickening within 500 µm of the fovea, accompanied by a decrease in visual acuity.

All patients were treated according to prevailing guidelines at the Ophthalmology Department of the Medical University of Innsbruck. The standard treatment protocol included a loading dose of three intravitreal injections of anti-VEGF (aflibercept—2.0 mg, 50 µL) administered monthly for three consecutive months, followed by additional injections every two months on a pro re nata basis. For cases where anti-VEGF therapy proved insufficient, intravitreal corticosteroids (40 mg/mL Triescence or 50 µL of Ozurdex) were administered.

The exclusion criteria are as follows: (1) the presence of age-related macular degeneration, (2) a history of retinal vein occlusion, (3) a history of uveitis, (4) a history of cardio-embolic events, (5) neurological diseases, (6) peripheral vascular diseases (e.g., diabetic foot or wound healing problems), and (7) diabetic nephropathy.

This study adhered to the principles outlined in the Declaration of Helsinki and was approved by the local ethics committee of the Medical University of Innsbruck (Innsbruck, Austria—No 1261/2020). Written informed consent was obtained from all participants after an explanation of this study’s nature and possible consequences.

### 4.2. Clinical Assessment

All patients were diagnosed and monitored through a follow-up by retinal specialists using fundoscopy and optical coherence tomography (OCT—Heidelberg Spectralis^®^ OCT, Heidelberg Engineering, Heidelberg, Germany). DR was classified into non-proliferative (NPDR) and proliferative (PDR).

Data were collected from an electronic database of patients receiving anti-VEGF therapy at the Department of Ophthalmology. The following information was recorded: visual acuity, expressed as the logarithm of the minimum-angle-of-resolution (logMAR) central macular thickness at baseline; the presence of bilateral disease; presence of PDR; the number of intravitreal injections of anti-VEGF and corticosteroids, which were given at the time of blood sample collection; signs of cataract; daily cigarette consumption; body mass index (BMI); the presence of hypertension; the type of diabetes; and patient demographic data, including age and sex. Blood samples were collected for biochemical analysis. Serum creatinine (Cr) and C-reactive protein (CRP) levels were measured to assess renal function and systemic inflammation, respectively.

### 4.3. RT-PCR of mRNA

Fasting whole blood samples were drawn and immediately centrifuged for 10 min at 3600× *g*. The buffy coat, containing lymphocytes, monocytes, and granulocytes, was extracted, resuspended in TRIzol reagent (Invitrogen, Waltham, MA, USA), and stored at −80 °C until analysis. Total RNA was extracted from fibroblasts using the TRIzol reagent (Invitrogen) according to the manufacturer’s instructions. RNA concentration and purity were measured using a nanophotometer (Implen, Westlake Village, CA, USA). Subsequently, reverse transcription of total mRNA into cDNA was performed using the QuantiTect RT kit(Qiagen GmbH, 40724 Hilden, Germany), following the manufacturer’s protocol.

For RT-PCR analysis of progerin, primers were designed and optimized to span the splice junction site between exon 11 and 12, as described in previous investigations by our study group. The progerin PCR product was confirmed by sequencing, demonstrating the progerin-specific gap of 150 bp between exon 11 and 12 in the blood [8,9].

Exon-spanning primers were verified on agarose gels (Figure 2). The amplification primers used are as follows:

GAPDH-F: 5′-GAGCCACATCGCTCAGACAC-3′

GAPDH-R: 5′-CATGTAGTTGAGGTCAATGAAGG-3′

RPL32-F: 5′-AGTTCCTGGTCCACAACGTC-3′

RPL32-R: 5′-CTCTTTCCACGATGGCTTTG-3′

LMNA spec F: 5′-TCAGGAGCCCAGAGCCCCCAGAAC-3′

LMNA spec R: 5′-GGGTTATTTTTCTTTGGCTTCA-3′

LMNA total F: 5′-GGTGGTGACGATCTGGGCT-3′

LMNA total R: 5′-CCAGTGGAGTTGATGAGAGC-3′

The cycling conditions for qPCR were 95 °C for 10 min (Activation), 95 °C for 15 s (Denaturation), and 60 °C for 1 min (Annealing and extension) up to 40 cycles. Using 2× SYBR green mastermix (Applied Biosystems, Waltham, MA, USA), quantitative gene expression was calculated by the comparative ΔΔCt method, with GAPDH as the reference gene. SYBR Green PCR was conducted using exon-spanning primers designed to ensure specific amplification. Standard cycling conditions were applied, and all runs included no-template controls. However, melt curve analysis and precise Ct value documentation were not performed, representing a limitation of the current methodology.

### 4.4. Immunostaining of Progerin in Dermal Fibroblasts

Dermal fibroblasts were obtained from two Hutchinson–Gilford Progeria Syndrome (HGPS) donors (an 8-year-old female and a 14-year-old male), provided by Coriell Institute for medical research. Human pulmonary artery adventitial fibroblasts (HPAAF) were provided by ScienCell Research Laboratories (cat. 3120). The cells were cultured in a fibroblast growth medium (Lonza, Morristown, NJ, USA) supplemented with 10% fetal calf serum (FCS) and 1% penicillin-streptomycin at 37 °C with 5% CO_2_ atmosphere. The cells were fixed in 4% paraformaldehyde for 10 min at room temperature, followed by permeabilization with 0.1% Triton X-100 in PBS for 5 min. After blocking with 5% bovine serum albumin (BSA) for 1 h, the cells were incubated overnight at 4 °C with a primary anti-progerin antibody (1:50 dilution) (13A4D4, Santa Cruz Biotechnology, Dallas, TX, USA). The next day, the cells were washed and incubated with a fluorescently labeled secondary antibody (Alexa Fluor 647, Merck Gesselschaft mbH, Vienna, Austria 1:1000 dilution) for 1 h at room temperature. The nuclei were counterstained with DAPI (1 µg/mL) for 5 min. Fluorescence microscopy was performed using an Olympus Vs120 microscope, Hamburg, Germany and images were captured to visualize progerin expression and nuclear localization.

### 4.5. Western Blot Analysis of Progerin

Fibroblast lysates were prepared by resuspending the cells in RIPA buffer containing protease inhibitors (Roche Diagnostics, Basel, Switzerland). Protein concentrations were determined using the Bradford assay (Bio-Rad, Hercules, CA, USA). Equal amounts of protein (50 µg) were loaded onto a 10% SDS-PAGE gel and transferred to a PVDF membrane (Millipore, Burlington, MA, USA). The membrane was blocked with 5% non-fat dry milk in TBS-Tween for 1 h at room temperature. Progerin was detected using a primary anti-progerin antibody (1:1000 dilution, 13A4D4, Santa Cruz Biotechnology) overnight at 4 °C, followed by incubation with an HRP-conjugated secondary antibody (1:5000 dilution) for 1 h at room temperature. TBP was used as a loading control. Bands were visualized using enhanced chemiluminescence (ECL, Thermo Fisher Scientific, Waltham, MA, USA), and densitometric analysis was performed.

### 4.6. Statistical Analysis

Statistical analysis was performed using SSPS™ Statistics 24.0.0 (IBM, Armonk, NY, USA), and GraphPad Prism (Version 6.04, GraphPad Software, Inc., San Diego, CA, USA) was used to generate graphics. The distribution of continuous variables was evaluated using the Kolmogorov–Smirnov test. Normally distributed continuous variables are reported as means with standard deviation (SD), whereas non-normally distributed variables are reported as the median with interquartile range (IQR). Categorical variables are presented as absolute values and percentages. Comparisons of categorical data between groups were performed using Fisher’s exact test. Normally distributed continuous data were compared using the independent *t*-test, while non-normally distributed data were analyzed using the Mann–Whitney U test. The tests were conducted in a two-tailed manner, and a *p*-value of less than 0.05 was considered significant. Correlation coefficients were determined by linear regression analysis (Pearson’s equation), and scatter plots were created. To account for the potential confounding effect of age on the relationship between progerin mRNA/GAPDH and clinical parameters, an age adjustment analysis was performed using multiple linear regression. For each dependent variable (IVOM anti-VEGF applications, macula thickness, and pack-years), we conducted a regression analysis, with age included as a covariate: Y_adjusted_ = Y_observed_ − (β_age_ × _Age_), where Yadjusted represents the age-adjusted residuals, Yobserved is the original clinical variable, and β_age_ is the regression coefficient for age. The residual values from these models were then used as age-adjusted clinical parameters, effectively removing the variance explained by age. These adjusted values were subsequently plotted against progerin mRNA/GAPDH expression levels. To assess whether age adjustment improved the association, we compared the pre- and post-adjustment regression models, reporting changes in R^2^ values and *p*-values.

## 5. Conclusions

In conclusion, this study provides evidence for the association of progerin expression with DME and premature aging in DR. Our findings demonstrate higher progerin mRNA levels in patients with NPDR compared to PDR and a positive correlation between progerin mRNA levels and clinical parameters such as the number of intravitreal anti-VEGF injections, CMT, and nicotine consumption, independent of age. These findings suggest that progerin may serve as a potential biomarker for DME and premature aging in DR.

Furthermore, this study highlights the role of progerin in premature aging and vascular damage, as evidenced by the elevated levels of progerin mRNA and protein in HGPS fibroblasts. The strong nuclear localization of progerin in fibroblasts further emphasizes its disruptive effect on the nuclear architecture, which has been previously associated with cellular dysfunction and aging. In summary, our findings suggest that progerin plays a role in the pathogenesis of DR, potentially contributing to premature aging and DME. Further research is needed to fully elucidate the mechanisms underlying progerin expression and its impact on DR, which may lead to the development of novel therapeutic strategies targeting progerin for the treatment of DR.

## Figures and Tables

**Table 1:** Demographic and clinical baseline characteristics.

Characteristics	
N (%)	140 (100)
Age (SD)	66 (12)
Bilateral pseudophakia	70 (50)
Female sex (%)	42 (30)
BMI	29 (4.5)
Hypertension (%)	101 (72)
Nicotine abuse (%)	45 (32)
Pack-years (IQR)	10 (0–38)
Type I diabetes (%)	28 (20)
HbA1c (SD)	7.8 (1.2)
IDDM (%)	112 (80)
Bilateral disease (%)	130 (93)
Onset of disease in years (IQR)	3.6 (1.3–6.9)
Proliferative DR (%)	53 (38)
N of anti-VEGF injections (IQR)	7 (2–24)
Serum creatine levels (Cr) (SD)	0.95 (0.29) mg/dL
CRP levels (SD)	4.36 (31.69) mg/dL
Visus logMAR (SD)	0.28 (0.29)

N—number of patients, BMI—body mass index, HbA1c—glycated hemoglobin, IDDM—insulin-dependent diabetes mellitus, DR—diabetic retinopathy, VEGF—vascular endothelial growth factor, and SD—standard deviation.

**Table 2:** Demographic and clinical baseline characteristics of patients with NPDR and patients with PDR.

Characteristics	NPDR	PDR	*p*-Value
N (%)	87 (62)	53 (38)	<0.001 *
Age (SD)	69 (10)	61 (12)	<0.001 *
Female sex (%)	26 (30)	16(30)	0.970
BMI (SD)	28 (4.6)	29 (4.1)	0.266
Hypertension (%)	63 (72)	38 (72)	0.990
Nicotine abuse (%)	33 (38)	12 (23)	0.360
Pack-years (IQR)	13 (0–40)	10 (0–21)	0.090
Type I diabetes (%)	13 (15)	15 (28)	0.138
HbA1c (SD)	7.6 (1.1)	8.0 (1.2)	0.133
IDDM (%)	65 (75)	47 (88)	0.231
Bilateral disease (%)	78 (90)	52 (98)	0.094
Onset of disease in years (IQR)	3.2 (1.1–6.6)	4.4 (1.8–7.2)	0.302
N of anti-VEGF injections (IQR)	10 (4–34)	5 (2–11)	0.035 *
Cataract OD (%)	19 (22)	12 (23)	0.973
Cataract OS (%)	22 (25)	13 (25)	0.919
Pseudophakia OD (%)	64 (74)	41 (77)	0.690
Pseudophakia OS (%)	65 (75)	40 (75)	0.999
Bilateral pseudophakia	47 (54)	40 (46)	0.378
Visus logMAR (SD)	0.27 (0.31)	0.29 (0.27)	0.788

N—number of patients, *—significant difference, BMI—body mass index, HbA1c—glycated hemoglobin, IDDM—insulin-dependent diabetes mellitus, DR—diabetic retinopathy, VEGF—vascular endothelial growth factor, OD—right eye, OS—left eye, and SD—standard deviation.

## Data Availability

The data presented in this study are available upon request from the corresponding author.
